# Possible Mechanisms of Di(2-ethylhexyl) Phthalate-Induced MMP-2 and MMP-9 Expression in A7r5 Rat Vascular Smooth Muscle Cells

**DOI:** 10.3390/ijms161226131

**Published:** 2015-12-04

**Authors:** Mei-Fen Shih, Kuang-Hung Pan, Jong Yuh Cherng

**Affiliations:** 1 Department of Pharmacy, Chia-Nan University of Pharmacy and Science, Tainan 717, Taiwan; meifenshih@mail.cnu.edu.tw; 2 Department of Biochemistry and Chemistry, National Chung Cheng University, Chia-Yi 621, Taiwan; s9923042@gmail.com

**Keywords:** di(2-ethylhexyl) phthalate (DEHP), vascular smooth muscle cells (VSMC), MMP-2, MMP-9, p38 MAPK, ERK1/2, Akt, NF-κB

## Abstract

Proliferation and migration of vascular smooth muscle cells (VSMC) are important in the development and/or progression of many cardiovascular diseases, including atherosclerosis. Evidence shows that matrix metalloproteinase (MMP)-2 and MMP-9 are related to the pathogenesis of atherosclerosis. The expressions of MMP-2 and MMP-9 in atherosclerosis are regulated via various pathways, such as p38 mitogen activated protein kinase (MAPK), extracellular signal regulated kinase 1 and 2 (ERK1/2), Akt, and nuclear factor kappa (NF-κB). Di(2-ethylhexyl) phthalate (DEHP) has been shown to induce atherosclerosis by increasing tumor necrosis factor (TNF)-α, interleukin (IL)-6, and intercellular adhesion molecule (ICAM) productions. However, whether DEHP poses any effects on MMP-2 or MMP-9 expression in VSMC has not yet been answered. In our studies, rat aorta VSMC was treated with DEHP (between 2 and 17.5 ppm) and p38 MAPK, ERK1/2, Akt, NF-κB, and MMP-2 and MMP-9 proteins and activities were measured. Results showed that the presence of DEHP can induce higher MMP-2 and MMP-9 expression than the controls. Similar results on MMP-regulating proteins, *i.e.*, p38 MAPK, ERK1/2, Akt, and NF-κB, were also observed. In summary, our current results have showed that DEHP can be a potent inducer of atherosclerosis by increasing MMP-2 and MMP-9 expression at least through the regulations of p38 MAPK, ERK1/2, Akt, and NF-κB.

## 1. Introduction

Proliferation and migration in smooth muscle cells (SMC) are known to involve formation of atherosclerosis [1]. Up-regulation of matrix metalloproteinase (MMP)-2 and MMP-9 has been identified in atherosclerotic plaques [2,3]. MMP-2 expression in vascular smooth muscle cells (VSMC) has been linked to several pathological situations, particularly in atherosclerotic plaques, which suggesting a pathogenic role for MMP-2 in the progression of atherosclerosis [4]. MMP-9 is also demonstrated to be critical for the development of arterial lesions by regulating both proliferation and migration of SMC in MMP^−/−^ mice [5]. Diverse signal transduction systems have been proposed to control VSMC proliferation, among them are mitogen activated protein kinase (MAPK), extracellular signal regulated kinase 1 and 2 (ERK1/2), Akt, and nuclear factor kappa (NF-κB) [1,6].

Di(2-ethylhexyl) phthalate (DEHP) is one of the most common compounds in environmental endocrine disruptors. DEHP is widely used as a plasticizer in most of plastic products, e.g., children’s toys, floor and wall coverings, food containers, baby bottles, blood storage bags, and medical devices [7,8]. Most studies show that DEHP affects both mouse and human reproductive systems [9,10]. Some studies even showed that DEHP is related to certain types of cancer development [11] and hepatotoxicity [12]. Recently, DEHP was shown to be associated with atherosclerosis-related cardiovascular disease complication by promoting low-density lipoprotein oxidation and increasing expression of pro-inflammatory mediators, such as tumor necrosis factor (TNF)-α, interleukin (IL)-6, and monocyte chemoattractant protein (MCP)-1 [13]. In addition, DEHP has been shown to stimulate mRNA and protein expression of intercellular adhesion molecule-1 (ICAM-1) in human umbilical vein endothelial cells [13,14]. However, effects of DEHP on VSMC are still not known. In this study, VSMC were treated with various dosages of DEHP. The activities as well as expression of MMP-2 and MMP-9 were measured by MMP activity ELISA kits and Western blotting, respectively. Their possible regular pathways, e.g., p38 MAKP, ERK1/2, Akt, and NF-κB, were all analyzed after the treatment of DEHP.

## 2. Results and Discussion

### 2.1. The Effects of Di(2-ethylhexyl)phthalate (DEHP) on Vascular Smooth Muscle Cells (VSMC) Cell Viability

**Figure 1:**
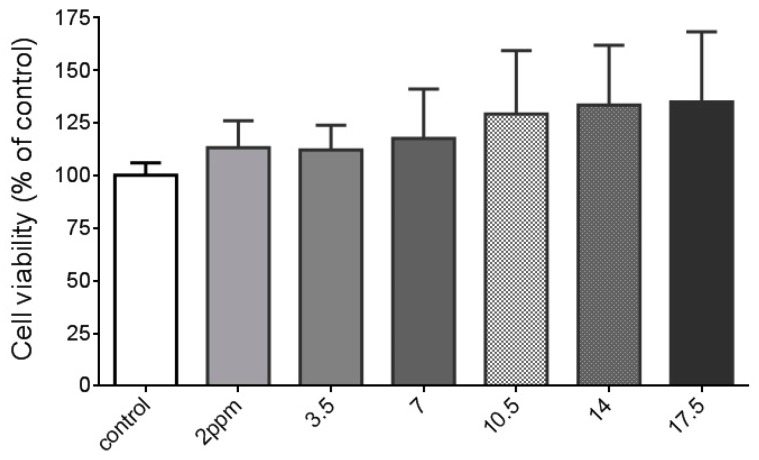
Effects of di(2-ethylhexyl) phthalate (DEHP) on cell viability in vascular smooth muscle cells (VSMC). VSMC (*n* ≥ 8) were treated with DEHP (concentrations between 2 and 17.5 ppm) for 24 h prior to cell viability being measured.

The tolerable daily intake of DEHP is 0.05 mg/kg body weight per day, according to European commission and Taiwan food and drug administration. Therefore, we chose 3.5 ppm (calculated as an adult with 70 kg body weight) as the basal level. We also use two, three, four, and five times higher doses (*i.e.*, 7, 10.5, 14, and 17.5 ppm) of DEHP to see any possible potentiated effects of DEHP on MMP-2 and MMP-9. A lower dose of DEHP (2 ppm) was also included in this study. Dosage of DEHP between 2 and 17.5 ppm did not cause any cell toxicity in A7r5 VSMC cells (Figure 1). Although some research showed that DEHP at concentrations more than 10 and 25 µM induced mouse Leydig cells cytotoxicity [15] and apoptosis in rat hepatocytes [12], respectively, no similar results have been reported in SMC or VSMC.

### 2.2. The Effects of DEHP on Matrix Metalloproteinase (MMP)-2 and -9 Protein Expression and Their Activities in the VSMC

**Figure 2:**
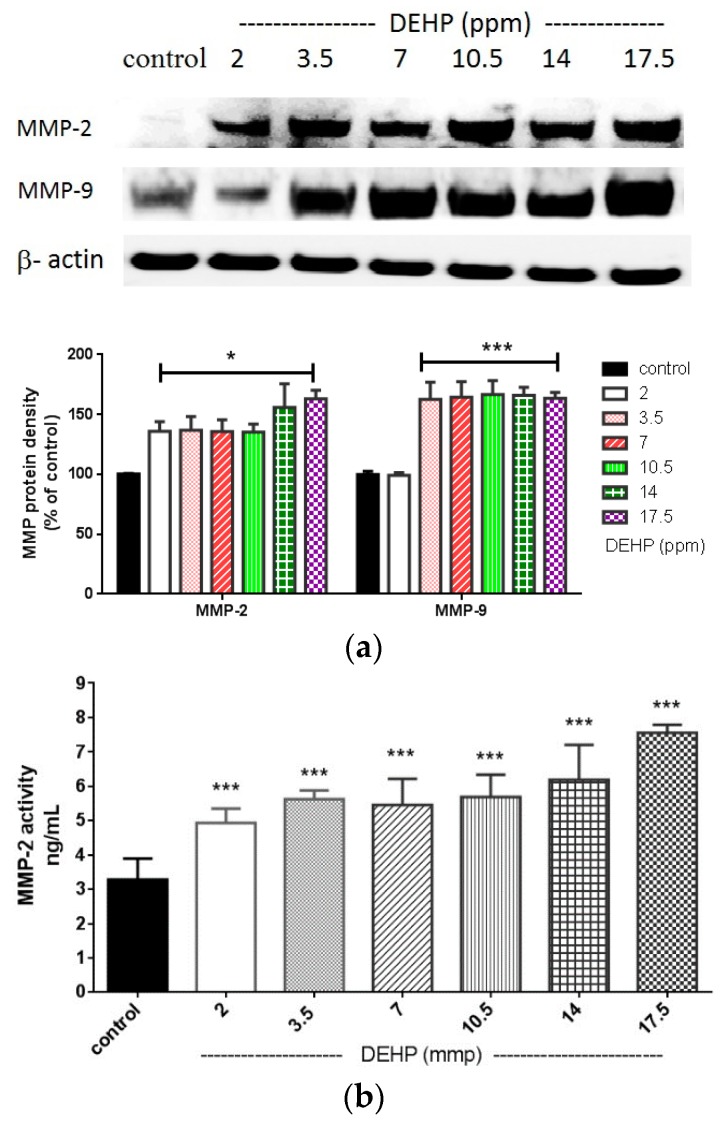

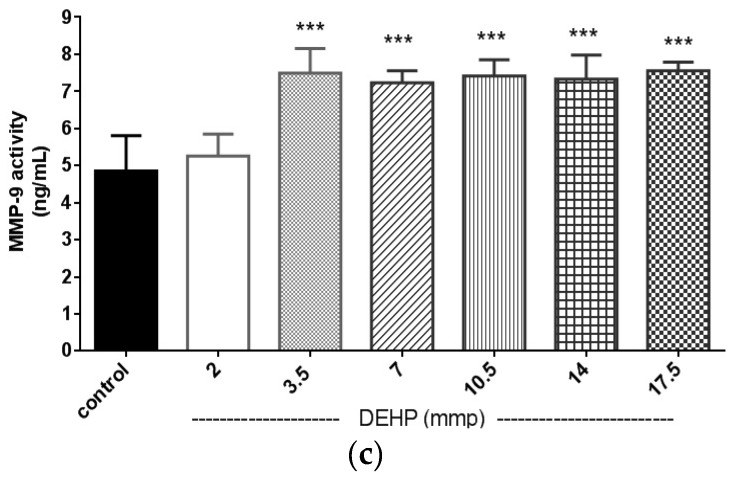
(**a**) Effects of DEHP on MMP-2 and MMP-9 protein expression. VSMC (*n* ≥ 3) were treated with DEHP (concentrations between 2 and 17.5 ppm) for 24 h prior to MMP-2 and MMP-9 protein extraction and expression. Statistics are shown for DEHP-treated cells * *p* < 0.05 and *** *p* < 0.005, compared to the respective controls; (**b**) Effects of DEHP on MMP-2 activity. VSMC were treated (*n* ≥ 8) with DEHP (concentrations between 2 and 17.5 ppm) for 24 h prior to MMP-2 activities being measured. Statistics are shown for DEHP-treated cells *** *p* < 0.005, compared to the control; and (**c**) Effects of DEHP on MMP-9 activity. VSMC were treated (*n* ≥ 8) with DEHP (concentrations between 2 and 17.5 ppm) for 24 h prior to MMP-9 activities being measured. Statistics are shown for DEHP-treated cells *** *p* < 0.005, compared to the control.

Major studies regarding to toxicities of DEHP are based on aspects other than atherosclerosis. A recent study showed that DEHP may accelerate atherosclerosis in apolipoprotein E-deficient mice [13]. However, the atherogenic effects of DEHP have not been studied in VSMC. In this study, the induction of MMP-9 expression by DEHP was observed at doses of 3.5 ppm and above (Figure 2a). DEHP-induced MMP-9 activities were also obtained between 3.5 and 17.5 ppm (Figure 2c). Surprisingly, DEHP could induce MMP-2 expression at dose as low as 2 ppm. The MMP-2 activities were also significantly induced by DEHP from 2 to 17.5 ppm (*p* < 0.005, Figure 2b).

Although DEHP showed to induce MMP-2 and MMP-9 by its active metabolite mono-(2-ethylhexyl) phthalate (MEHP) in human testicular embryonal carcinoma cells [16], the DEHP-induced MMP-2 and MMP-9 activities and protein expression in VSMC in relation to atherosclerosis were barely investigated [13]. Moreover, the inductive effects of MEHP on MMP-2 and MMP-9 may not be completely responsible for the observed DEHP impact on MMP-2 and MMP-9, since DEHP could still remain in the circulation for some time before it is all metabolized to MEHP and other metabolites (e.g., 5-OH-MEHP and 5-oxo-MEHP). Therefore, the results shown indicate that DEHP could still be a potential atherogenic substance *in vivo*. MMP-9 has been indicated as a prognostic biomarker for individuals at increased risk of cardiovascular mortality [17]. MMP-9 is also associated with post myocardial infarction cardiac remodeling, inflammation, and left ventricular wall rupture [18]. The action of MMP-9 on the development of atherosclerosis has been demonstrated in Apo E/MMP-9 double-null mice, which developed less atherosclerosis progression compared to Apo E null/MMP-9^+/+^ mice with a high fat diet for eight weeks [19]. In addition, MMP-9 is critical for the development of arterial lesions by regulating both SMC proliferation and migration, which was demonstrated in a MMP-9^−/−^ mice model [5]. Although DEHP at dosage of 3.5 ppm is considered as a safe dose for an adult in general aspects, our results indicated that it is able to induce the expression of MMP-9 noticeably. This means that DEHP is a possible risk factor for developing atherosclerosis and other CV diseases after long term exposure. Moreover, evidence showed that the ratio of MMP-2 expression in atherosclerotic plaque/fatty streak/normal aortic wall was approximately 4:2:1, respectively [20]. In addition, higher MMP-2 activity was shown in SMC-rich lesion [21]. Our data showed that DEHP induced MMP-2 activity and protein expression in VSMC at a dose as low as 2 ppm. Results from DEHP-induced MMP-2 and MMP-9 expression (Figure 2a) and activities (Figure 2b,c) indicate that DEHP is a very potent promoter for the migration of VSMC, which leads an important progression of atherosclerosis.

### 2.3. Regulatory Pathways of MMP-2 and MMP-9 by DEHP via Phosphorylated p38 Mitogen Activated Protein Kinase (MAPK), Phosphorylated Extracellular Signal Regulated Kinase 1 and 2 (ERK1/2), Phosphorylated Akt, and Nuclear Factor Kappa (NF-κB) (p65)

**Scheme 1:**
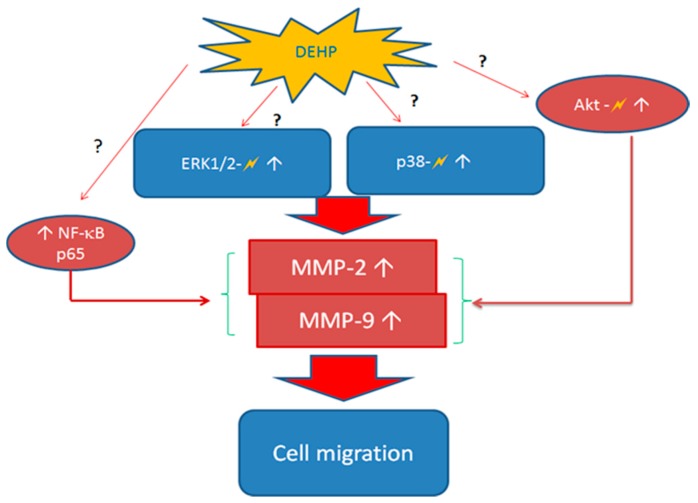
Possible mechanisms of DEHP induced MMP-2 and MMP-9 activities and protein expression. Pathways with question marks are clarified in this study. White arrows indicate an increase in activity or expression of proteins. Lighting signs indicate that proteins are activated (phosphorylated).

**Figure 3:**
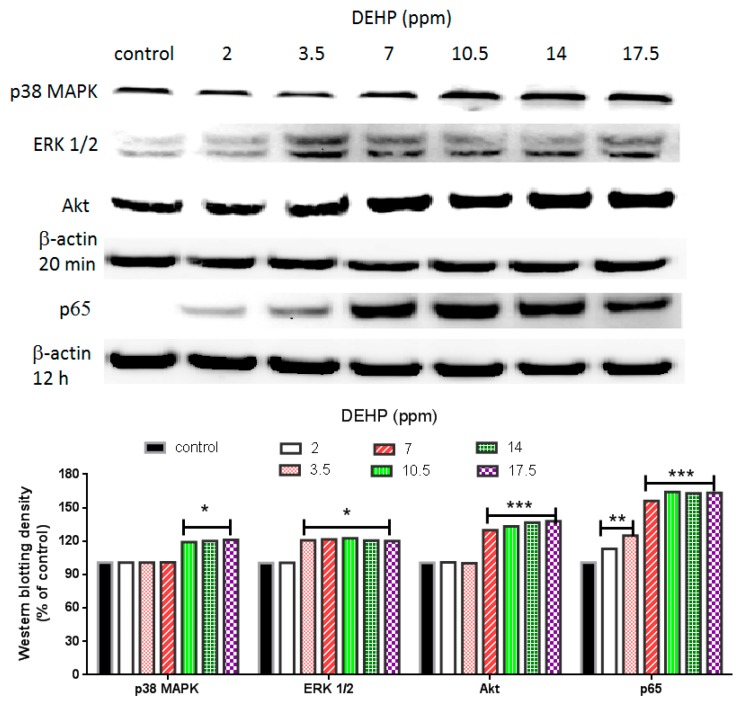
Effects of DEHP on phosphorylated p38 MAPK, phosphorylated ERK1/2, phosphorylated Akt, and NF-κB (p65) protein expression. VSMC (*n* ≥ 3) were treated with DEHP (concentrations between 2 and 17.5 ppm) for 20 min (p38 MAPK, ERK1/2, and Akt) or 12 h (NF-κB) prior to protein extraction. Phosphorylated p38 MAPK, phosphorylated ERK1/2, phosphorylated Akt, and NF-κB (p65) were expressed by Western blotting. Statistics are shown for DEHP-treated cells * *p* < 0.05, ** *p* < 0.01, and *** *p* < 0.005, compared to the respective control groups.

**Figure 4:**
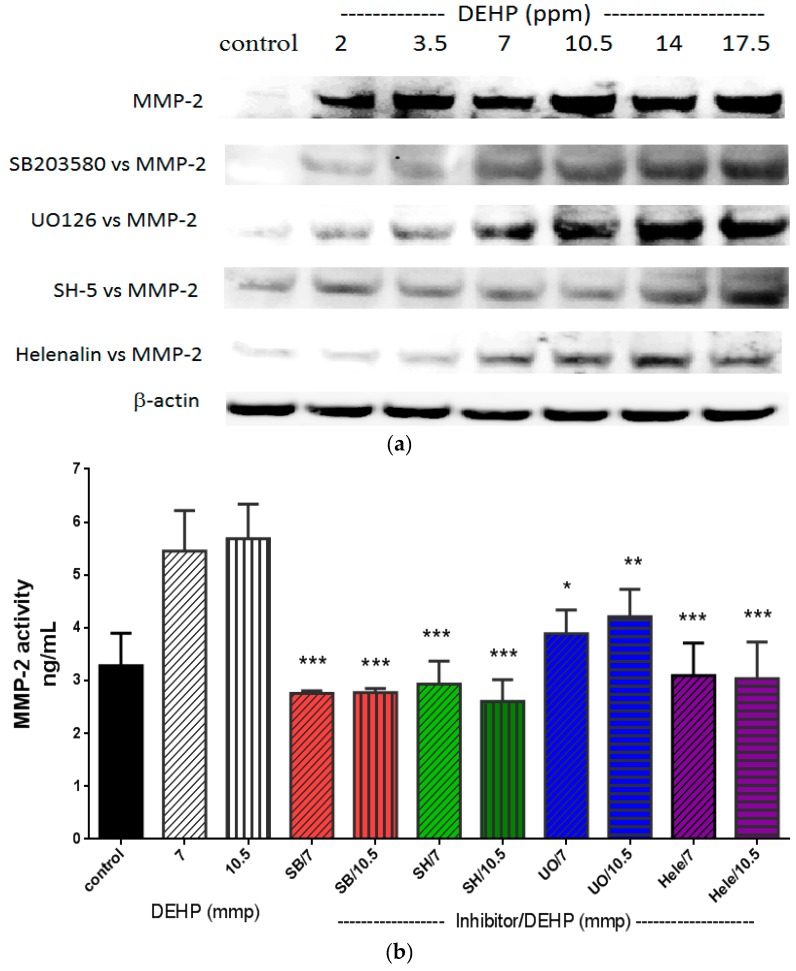
(**a**) Effects of p38 MAPK, ERK1/2, Akt, and NF-κB inhibitors on DEHP-induced MMP-2 expression. VSMC (*n* ≥ 3) were pre-treated with SB203580 (10 µM), UO126 (10 µM), SH-5 (10 µM), and Helenalin (10 µM) for 2 h, DEHP (concentrations between 2 and 17.5 ppm) was then added and incubated for further 24 h prior to protein extraction. MMP-2 was expressed by Western blotting; and (**b**) effects of p38 MAPK, ERK1/2, Akt, and NF-κB inhibitors on DEHP-induced MMP-2 activity. VSMC (*n* ≥ 3) were pre-treated with SB203580 (10 µM), UO126 (10 µM), SH-5 (10 µM), and Helenalin (10 µM) for 2 h, DEHP (7 or 10.5 ppm) was then added and incubated for further 24 h prior to MMP-2 activity being measured. Statistics are shown for all inhibitors-treated cells * *p* < 0.05, ** *p* < 0.01, and *** *p* < 0.005, compared to the DEHP-treated group.

**Figure 5:**
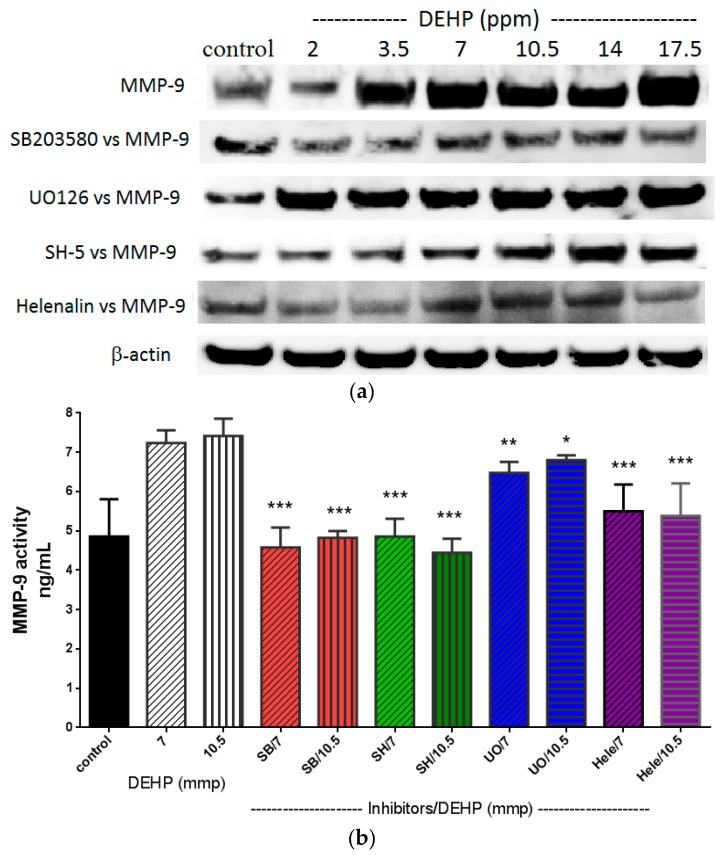
(**a**) Effects of p38 MAPK, ERK1/2, Akt, and NF-κB inhibitors on DEHP-induced MMP-9 expression. VSMC (*n* ≥ 3) were pre-treated with SB203580 (10 µM), UO126 (10 µM), SH-5 (10 µM), and Helenalin (10 µM) for 2 h, DEHP (between 2 and 17.5 ppm) was then added and incubated for further 24 h prior to protein extraction. MMP-9 was expressed by Western blotting; and (**b**) effects of p38 MAPK, ERK1/2, Akt, and NF-κB inhibitors on DEHP-induced MMP-9 activity. VSMC (*n* ≥ 3) were pre-treated with SB203580 (10 µM), UO126 (10 µM), SH-5 (10 µM), and Helenalin (10 µM) for 2 h, DEHP (7 or 10.5 ppm) was then added and incubated for further 24 h prior to MMP-9 activities being measured. Statistics are shown for SB203580-, SH-5-, and Helenalin-treated cells *** *p* < 0.005, compared to the DEHP-treated group; for UO126-treated cells (UO/7); ** *p* < 0.01, compared to the 7 ppm of DEHP-treated group; and for UO126-treated cells (UO/10.5); * *p* < 0.01, compared to the 10.5 ppm of DEHP-treated group.

The most common regulatory pathways of MMP-2 and MMP-9 are p38 MARK, ERK1/2, Akt, and NF-κB [22,23] (also see Scheme 1). DEHP induced NF-κB (p65) and phosphorylated ERK1/2 expression was observed at a dose as low as 2 and 3.5 ppm, respectively (Figure 3), whereas DEHP-induced phosphorylated Akt and phosphorylated p38 MAPK expression was observed at doses above 7 ppm of DEHP. Therefore, phosphorylated ERK1/2 and NF-κB (p65) seemed to play initial activation of MMP-2 and MMP-9. In order to clarify which pathway(s) plays the major role in regulating MMP-2 and MMP-9, we then used SB203580 [24], UO126 [25], SH-5 [25], and Helenalin [26], which are specific inhibitors of p38 MAKP, ERK1/2, Akt, and NF-κB, respectively. We found that the DEHP-induced MMP-2 expression was prevented by pre-treatment of SB203580 (p38 MAPK inhibitor), SH-5 (Akt inhibitor) and Helenalin (NF-κB inhibitor) (Figure 4a). Similar results were also observed in DEHP-induced MMP-9 expression (Figure 5a). However, the DEHP-induced MMP-2 expression was only partially affected by UO126 (ERK1/2 inhibitor). When doses of DEHP were higher than 7 ppm, the inhibitory effects of UO126 were diminished (Figure 4a). Alike, UO126 showed a slightly effect on DEHP-induced MMP-9 expression (Figure 5a).

For analysis of MMP-2 (Figure 4b) and MMP-9 (Figure 5b) activities under DEHP stimulation, 7 and 10.5 ppm were measured since higher doses of DEHP (≥7 ppm) could induce expression of all regulatory factors (p38 MAKP, ERK1/2, Akt, and NF-kB). Inhibitions of SB203580, SH-5, UO126, and Helenalin on DEHP-induced MMP-2 and MMP-9 activities were similar to MMP-2 and MMP-9 protein expression shown in Figure 4a,b, respectively. Although UO126 did not prevent 7 and 10.5 ppm of DEHP-induced MMP-2 and MMP-9 as strong as other inhibitors, MMP-2 (*t* < 0.05 and *t* < 0.01) and MMP-9 (*t* < 0.01 and *t* < 0.05) activities were still lower in UO126-treated cells than 7 and 10.5 ppm of DEHP-treated cells. Research showed that there could be a possible cross-talk between p38 MAPK and ERK1/2 in regulating MMP-2 [27] and MMP-9 [28]. Therefore, in our study, although the phosphorylated ERK1/2 expression was initially increased in VSMC by DEHP at a lower dose than phosphorylated p38 MAPK, pretreatment of ERK1/2 inhibitor did not block MMP-2 and MMP-9 expression at high doses of DEHP could be due to the cross-talk of certain regulatory pathways.

### 2.4. The Effects of DEHP on VSMC Migration

**Figure 6:**
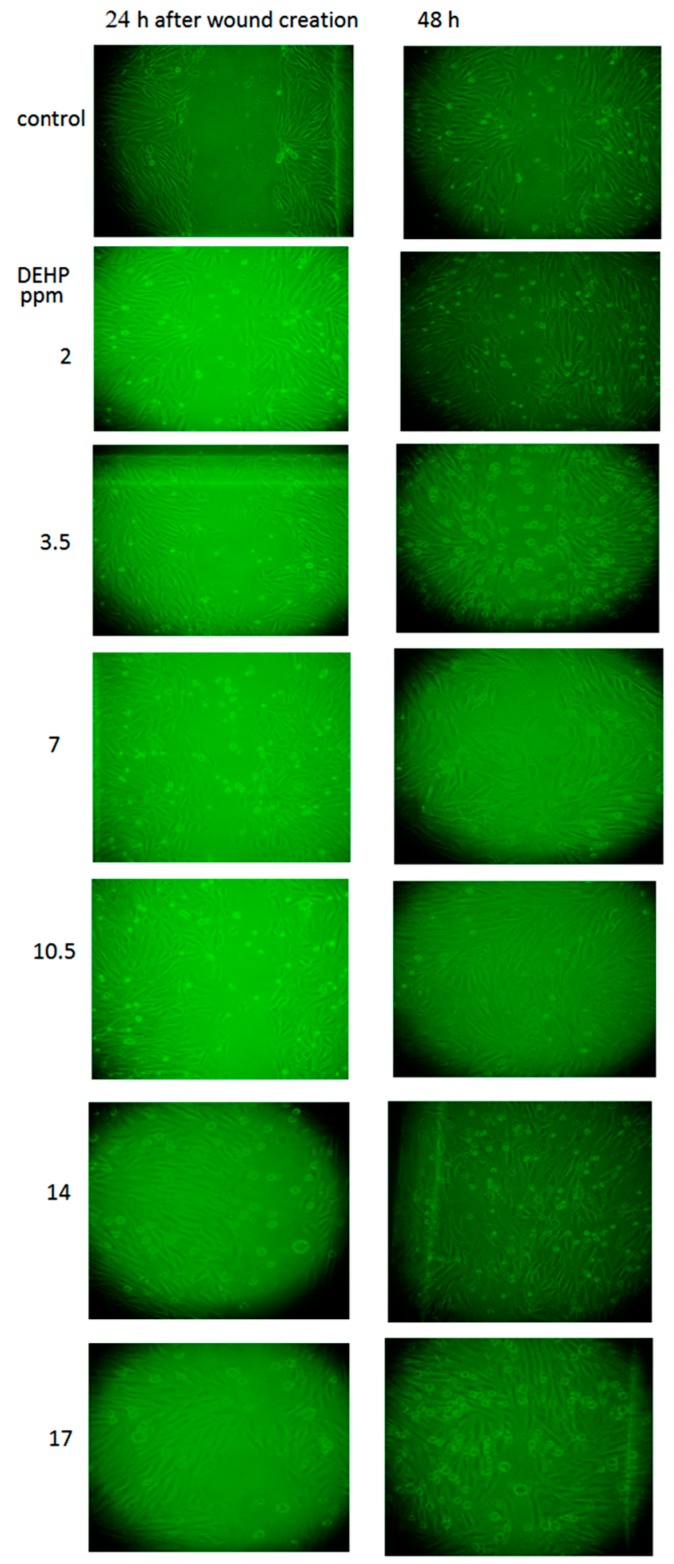
Effects of DEHP on cell migration. VSMC (*n* ≥ 3) were treated with DEHP (between 2 and 17.5 ppm) after the wound being created. Cell migration was observed under a light microscope (200×) at 24 h and 48 h after initial wound creation.

Cardiovascular disorders is often related to the abnormal proliferation and migration of VSMC in arterial walls, which has been reported to play pivotal roles in the initiation and progression of arteriosclerosis [29,30]. Our results showed that DEHP at doses higher than 3.5 ppm would induce cell migration 24 h and 48 h after the initial wound creation (Figure 6), whereas the DEHP-induced cell migration was inhibited by the presence of p38 MAPK, Akt or NF-κB inhibitor. By contrast, the migration was less affected by the treatment of ERK1/2 inhibitor (UO-126; shown as Figure S1). Taken together, all these results obtained in this study indicated that DEHP is potent inducer of atherosclerosis.

## 3. Experimental Section

### 3.1. Reagents

Cell proliferation reagent WST-1 (11644807001) was obtained from Roche (Taipei, Taiwan). Mouse anti-MMP-2 (35-1300Z), Anti-Akt/PKB “pT308” (44-602G), Anti-ERK 1 and 2 “pTpY185/187” (44680G), and anti-NF-κB (p65) (#436700) antibodies were obtained from Invitrogen. p38 MAPK “pTpY180/182” (#701057) was obtained from Life technology (Taipei, Taiwan). Rat anti-MMP-9 (STJ24592), was obtained from St John’s Laboratory (Taipei, Taiwan). Mammalian protein extraction reagent (#78501) was purchased from Thermo Scientific (Taipei, Taiwan). DEHP was obtained from Sigma (80030 Fluka) (Taipei, Taiwan). MMP-2 (RPN 2631) and MMP-9 (RPN 2634) Biotrak-Activity-Assay kits were purchased from GE Healthcare Life Science (Taipei, Taiwan). UO126 (#9903) and SB203580 (#5633) were obtained from Cell Signaling (Taipei, Taiwan). SH-5 (ab414142) was purchased from Abcam. Helenalin (#17050) was purchased from Cayman (Taipei, Taiwan).

### 3.2. Cell Cultures

A7r5 cells, smooth muscle cells of thoracic aorta (BCRC 60153) from rat, were obtained from Bioresource Collection and Research Center (Hsinchu, Taiwan). These cells were cultured in Eagle-MEM supplemented with 4 mM l-glutamine and 10% FBS and were maintained at 37 °C in a humidified atmosphere of 5% CO_2_–95% air. When the cells reached above 80% confluence, subculture was performed at a split ratio of 1:3.

### 3.3. Cell Viability and MMP-2 and MMP-9 Activity Assay

A7r5 (1 × 10^5^/well) were seeded in a 96-well plate. When the cell confluence reached above 85%–90%, various concentrations of DEHP (between 2 and 17.5 ppm) were mixed with 1% FBS culture medium and incubated for 24 h before cell viability was measured and the culture media were collected for MMPs activity assay. Stock of DEHP (5 mg/mL) was dissolved in methanol, the final concentration of methanol added into cells was <0.5%. The inhibitors of p38 MAPK (SB203580), ERK1/2 (UO126), Akt (SH-5), or NF-κB (Helenalin) were applied to the cells 2 h prior to DEHP treatment.

### 3.4. Western Blotting Analysis of MMP-2, MMP-9, p38 MAPK, ERK1/2, Akt and NF-κB Expression

A7r5 cells at a density of 5 × 10^5^ cells/well were cultured in 10 cm Petri dishes and awaited cells to reach 90%–95% confluence. A7r5 cells were then exposed to DEHP for 20 min (for p38 MAPK, ERK1/2, Akt), 12 h (for NF-κB), or 24 h (for MMP-2 and -9). After that, these cells were harvested and lysed with mammalian protein extraction reagent. The inhibitors of p38 MAPK (SB203580), ERK1/2 (UO126), Akt (SH-5), or NF-κB (Helenalin) were applied to the cells 2 h prior to DEHP treatment. Approximately 40 µg of cell lysate was boiled at 95 °C for 5 min in the sample buffer. The samples were then separated by 10% SDS-PAGE, and then protein blotted onto PVDF membranes. The protein-blotted membranes were blocked with 5% (*w*/*v*) fat-free dry milk in 0.05% Tween 20 (PBS-T) at 4 °C for overnight. They were then reacted with anti-MMP-2 or anti-MMP-9, anti-p38 MAPK, anti-ERK1/2, anti-Akt, anti-NF-κB or housekeeping β-actin antibody at 1:1000 dilution in PBS-T containing 1% bovine serum albumin overnight at 4 °C. After washing three times with PBS-T solution, blots were further incubated for 1 h at room temperature with goat anti-mouse IgG antibody coupled to horseradish peroxidase at 1:2000 dilution in PBS-T with 5% skim milk and washed three times (with PBS-T) before visualization. The expression of the proteins was detected by an ECL detection system.

### 3.5. Cell Migration Assay

A7r5 cells at a density of 2 × 10^5^ cells/well were seeded in 6 cm Petri dishes and awaited to reach 90%–95% cell confluence. Then, the surface of cell dishes was scratched with a sterile pipette tip and rinsed three times. Cells were incubated with or without DEHP (see above), for 24 and 48 h and then imaged using a Nikon Eclipse TS100 microscope (Nikon, Taipei, Taiwan).

### 3.6. Statistical Analysis

For cell viability, MMP-2 and MMP-9 activity assays, data from each group (*n* ≥ 8) in different experimental days were analyzed. A two-tailed student’s unpaired test was applied to compare the mean values of two populations of continuous data. Western blotting data were performed (*n* ≥ 3) and a representative one was shown.

## 4. Conclusions

This is the first time that the influence of DEHP on MMP-2 and MMP-9 expression and activities in VSMC were studied. Our results strongly suggest that DEHP may induce atherosclerosis development by increasing expression of MMP-2 and MMP-9 via p38 MAPK-, ERK1/2-, Akt-, and NF-κB-mediated pathways.

## Supplementary Materials

Supplementary materials can be found at http://www.mdpi.com/1422-0067/16/12/26131/s1.
