# Supplementary material for: Possible Mechanisms of Di(2-ethylhexyl) Phthalate-Induced MMP-2 and MMP-9 Expression in A7r5 Rat Vascular Smooth Muscle Cells
Source: Int J Mol Sci. 2015 Dec 4;16(12):28800–11. doi: 10.3390/ijms161226131 (PMC4691078; doi:10.3390/ijms161226131)
Supplement: Supplementary file 1 [file ijms-16-26131-s001.pdf]

Supplementary Materials: Possible Mechanisms of Di(2-ethylhexyl) Phthalate-Induced MMP-2 and MMP-9 Expression in A7r5 Rat Vascular Smooth Muscle Cells

Mei-Fen Shih, Kuang-Hung Pan and Jong Yuh Cherng

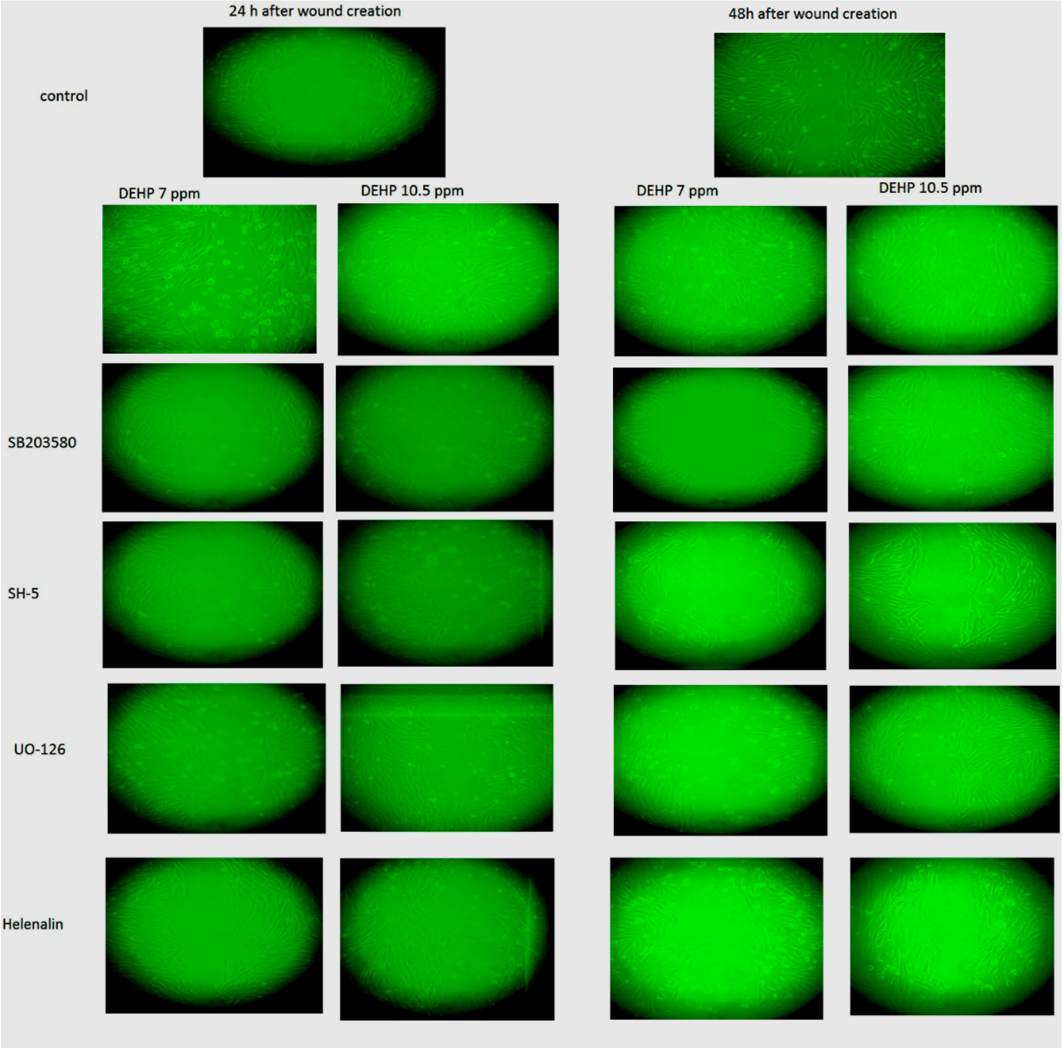

Figure S1. Effects of p38 MAPK inhibitor (SB203580), Akt inhibitor (SH-5), ERK1/2 inhibitor (UO126) and NF-κB inhibitor (Helenalin) on DEHP-induced VSMC migration. Cell migration was observed under a light microscope (200×) at 24 h and 48 h after initial wound creation.
